# Supplementary material for: Yeast Associated with Rice Phylloplane and Their Contribution to Control of Rice Sheath Blight Disease
Source: Microorganisms. 2020 Mar 3;8(3):362. doi: 10.3390/microorganisms8030362 (PMC7142645; doi:10.3390/microorganisms8030362)
Supplement: Supplementary file 1 [file microorganisms-08-00362-s001.pdf]

Table S1. Sequence similarity analysis of the D1/D2 domain of rice phylloplane yeasts and their closely related species.

Yeast strain	GenBank accession no.	Closest species	Nucleotide substitutions/gap/ total nt	% identity	Identification
DMKU-RP02	LC486512	*Saitozyma flava* (AF075497)	0/0/581	100	*Saitozyma flava*
DMKU-RP03	LC498132	*Debaryomyces nepalensis* (U45839)	0/0/524	100	*Debaryomyces nepalensis*
DMKU-RP04	LC498133	*Wickerhamomyces anomalus* (U74592)	0/0/479	100	*Wickerhamomyces anomalus*
DMKU-RP06	LC498134	*Kodamaea ohmeri* (U45702)	0/0/441	100	*Kodamaea ohmeri*
DMKU-RP07	LC498135	*Meyerozyma caribbica* (AY187283)	0/0/476	100	*Meyerozyma caribbica*
DMKU-RP08	LC498136	*Rhodotorula taiwanensis* (GU646863)	0/0/525	100	*Rhodotorula taiwanensis*
DMKU-RP09	LC498137	*Dirkmeia churashimaensis* (AB548955)	0/0/507	100	*Dirkmeia churashimaensis*
DMKU-RP11	LC486506	*Rhodotorula taiwanensis* (GU646863)	2/0/538	99.6	*Rhodotorula taiwanensis*
DMKU-RP12	LC498138	*Moesziomyces antarcticus* (KY108571)	0/0/545	100	*Moesziomyces antarcticus*
DMKU-RP14	LC498139	*Candida tropicalis* (U45749)	0/0/570	100	*Candida tropicalis*
DMKU-RP15	LC498140	*Candida tropicalis* (U45749)	0/0/530	100	*Candida tropicalis*
DMKU-RP16	LC498141	*Candida maltosa* (U45745)	0/0/452	100	*Candida maltosa*
DMKU-RP18	LC498142	*Kodamaea ohmeri* (U45702)	0/0/444	100	*Kodamaea ohmeri*
DMKU-RP20	LC498143	*Papiliotrema aspenensis* (KC469778)	1/0/514	99.0	*Papiliotrema aspenensis*
DMKU-RP22	LC498144	*Moesziomyces antarcticus* (KY108571)	0/0/545	100	*Moesziomyces antarcticus*
DMKU-RP23	LC498145	*Candida maltosa* (U45745)	0/0/452	100	*Candida maltosa*
DMKU-RP24	LC498146	*Kodamaea ohmeri* (U45702)	1/0/447	99.8	*Kodamaea ohmeri*
DMKU-RP25	LC498147	*Wickerhamomyces anomalus* (U74592)	1/0/527	99.8	*Wickerhamomyces anomalus*
DMKU-RP26	LC498148	*Meyerozyma guilliermondii* (U45709)	3/0/471	99.4	*Meyerozyma guilliermondii*
DMKU-RP27	LC498149	*Dirkmeia churashimaensis* (AB548955)	1/0/546	99.8	*Dirkmeia churashimaensis*
DMKU-RP28	LC498150	*Moesziomyces antarcticus* (KY108571)	0/0/545	100	*Moesziomyces antarcticus*
DMKU-RP29	LC486509	*Rhodotorula toruloides* (AF070426)	5/0/556	99.1	Potential new species closest to *Rhodotorula toruloides*
DMKU-RP30	LC498151	*Occultifur plantarum* (LC158346)	0/0/427	100	*Occultifur plantarum*
DMKU-RP31	AB920307	*Torulaspora indica* (NG_058414)	2/0/516	99.6	*Torulaspora indica*
DMKU-RP32	LC498152	*Rhodotorula taiwanensis* (GU646863)	0/1/474	99.8	*Rhodotorula taiwanensis*
DMKU-RP33	LC498153	*Symmetrospora vermiculata* (AF460176)	1/0/553	99.8	*Symmetrospora vermiculata*
DMKU-RP34	LC498154	*Kodamaea ohmeri* (U45702)	2/1/449	99.3	*Kodamaea ohmeri*
DMKU-RP35	LC498155	*Torulaspora indica* (NG_058414)	1/0/458	99.8	*Torulaspora indica*
DMKU-RP37	LC486507	*Rhodotorula taiwanensis* (GU646863)	2/1/503	99.4	*Rhodotorula taiwanensis*
DMKU-RP38	LC486508	*Rhodtorula toruloides* (AF070426)	3/1/559	99.3	*Rhodtorula toruloides*
DMKU-RP39	LC498156	*Moesziomyces parantarcticus*(AB089357)	0/0/526	100	*Moesziomyces parantarcticus*
DMKU-RP40	LC498157	*Hannaella sinensis* (NG_042362)	0/0/549	100	*Hannaella sinensis*
DMKU-RP42	LC498158	*Meyerozyma caribbica* (AY187283)	0/0/476	100	*Meyerozyma caribbica*
DMKU-RP44	LC498159	*Kodamaea ohmeri* (U45702)	0/0/436	100	*Kodamaea ohmeri*
DMKU-RP45	AB920308	*Hannaella sinensis* (NG_042362)	0/0/531	100	*Hannaella sinensis*
DMKU-RP47	LC498160	*Meyerozyma caribbica* (AY187283)	0/0/476	100	*Meyerozyma caribbica*
DMKU-RP48	LC498161	*Occultifur plantarum* (LC158346)	0/0/469	100	*Occultifur plantarum*
DMKU-RP49	LC498162	*Meyerozyma caribbica* (AY187283)	0/0/373	100	*Meyerozyma caribbica*

Table S1. *continued.*

Yeast strain	GenBank accession no.	Closest species	Nucleotide substitutions/gap/ total nt	% identity	Identification
DMKU-RP50	LC498163	*Rhodtorula toruloides* (AF070426)	0/0/393	100	*Rhodtorula toruloides*
DMKU-RP51	LC498164	*Meyerozyma caribbica* (AY187283)	0/0/373	100	*Meyerozyma caribbica*
DMKU-RP52	LC498165	*Dirkmeia churashimaensis* (AB548955)	0/0/545	100	*Dirkmeia churashimaensis*
DMKU-RP53	LC486510	*Rhodtorula toruloides* (AF070426)	5/0/577	99.1	Potential new species closest to *Rhodotorula toruloides*
DMKU-RP54	LC498166	*Hannaella sinensis* (NG_042362)	0/0/445	100	*Hannaella sinensis*
DMKU-RP55	LC498167	*Meyerozyma caribbica* (AY187283)	0/1/477	99.8	*Meyerozyma caribbica*
DMKU-RP57	LC498168	*Kodamaea ohmeri* (U45702)	0/0/469	98.9	*Kodamaea ohmeri*
DMKU-RP58	LC498169	*Symmetrospora vermiculata* (AF460176)	1/0/553	99.8	*Symmetrospora vermiculata*
DMKU-RP61	LC498170	*Rhodotorula taiwanensis* (GU646863)	0/1/556	99.8	*Rhodotorula taiwanensis*
DMKU-RP62	LC498171	*Dirkmeia churashimaensis* (AB548955)	0/0/507	100	*Dirkmeia churashimaensis*
DMKU-RP63	LC498172	*Moesziomyces antarcticus* (KY108571)	0/2/583	99.7	*Moesziomyces antarcticus*
DMKU-RP64	LC498173	*Meyerozyma caribbica* (AY187283)	1/0/471	99.8	*Meyerozyma caribbica*
DMKU-RP66	LC498174	*Moesziomyces antarcticus* (KY108571)	0/0/545	100	*Moesziomyces antarcticus*
DMKU-RP68	LC498175	*Rhodotorula mucilaginosa* (AF070432)	1/0/563	99.8	*Rhodotorula mucilaginosa*
DMKU-RP70	LC498176	*Sporobolomyces blumeae* (AY213010)	1/0/528	99.8	*Sporobolomyces blumeae*
DMKU-RP71	LC498177	*Moesziomyces antarcticus* (KY108571)	1/1/581	99.7	*Moesziomyces antarcticus*
DMKU-RP72	NG_060619	*Hannaella siamensis* (AB922844)	0/0/597	100	*Hannaella siamensis*[a]
DMKU-RP74	LC498265	*Moesziomyces antarcticus* (KY108571)	0/0/611	100	*Moesziomyces antarcticus*
DMKU-RP76	LC498266	*Pseudozyma hubeiensis* (DQ008953)	0/0/566	100	*Pseudozyma hubeiensis*
DMKU-RP77	LC498267	*Hannaella phetchabunensis* (AB922849)	0/0/597	100	*Hannaella phetchabunensis*
DMKU-RP79	LC498268	*Dirkmeia churashimaensis* (AB548955)	1/0/588	99.8	*Dirkmeia churashimaensis*
DMKU-RP80	LC498269	*Moesziomyces antarcticus* (KY108571)	1/1/591	99.7	*Moesziomyces antarcticus*
DMKU-RP81	LC498270	*Dirkmeia churashimaensis* (AB548955)	0/0/577	100	*Dirkmeia churashimaensis*
DMKU-RP82	LC498271	*Moesziomyces antarcticus* (KY108571)	0/0/611	100	*Moesziomyces antarcticus*
DMKU-RP83	LC498272	*Rhodotorula mucilaginosa* (AF070432)	0/0/488	99.6	*Rhodotorula mucilaginosa*
DMKU-RP84	LC498273	*Rhodotorula taiwanensis* (GU646863)	1/0/574	99.8	*Rhodotorula taiwanensis*
DMKU-RP85	LC498274	*Sporidiobolus pararoseus* (AF070437)	0/0/535	100	*Sporidiobolus pararoseus*
DMKU-RP86	LC498275	*Dirkmeia churashimaensis* (AB548955)	1/1/579	99.7	*Dirkmeia churashimaensis*
DMKU-RP88	LC498276	*Papiliotrema rajasthanensis* (AM262324)	1/1/565	99.6	*Papiliotrema rajasthanensis*
DMKU-RP89	LC498277	*Rhodotorula taiwanensis* (GU646863)	1/4/543	99.1	*Rhodotorula taiwanensis*
DMKU-RP90	LC498278	*Dirkmeia churashimaensis* (AB548955)	0/1/532	99.8	*Dirkmeia churashimaensis*
DMKU-RP91	LC498279	*Meyerozyma caribbica* (AY187283)	0/0/525	100	*Meyerozyma caribbica*
DMKU-RP93	LC498280	*Moesziomyces antarcticus* (KY108571)	0/0/569	100	*Moesziomyces antarcticus*
DMKU-RP94	LC498281	*Rhodotorula taiwanensis* (GU646863)	1/3/541	99.3	*Rhodotorula taiwanensis*
DMKU-RP96	LC498282	*Rhodotorula paludigena* (AF070424)	0/0/539	100	*Rhodotorula paludigena*
DMKU-RP97	LC498283	*Moesziomyces antarcticus* (KY108571)	0/1/585	100	*Moesziomyces antarcticus*
DMKU-RP98	LC498284	*Sporobolomyces blumeae* (AY213010)	0/0/546	100	*Sporobolomyces blumeae*
DMKU-RP99	LC498285	*Papiliotrema rajasthanensis* (AM262324)	0/2/561	99.6	*Papiliotrema rajasthanensis*

Table S1. *continued.*

Yeast strain	GenBank accession no.	Closest species	Nucleotide substitutions/gap/ total nt	% identity	Identification
DMKU-RP100	LC498286	*Moesziomyces antarcticus* (KY108571)	1/0/569	99.8	*Moesziomyces antarcticus*
DMKU-RP101	LC498287	*Moesziomyces antarcticus* (KY108571)	0/2/576	99.7	*Moesziomyces antarcticus*
DMKU-RP103	LC498288	*Dirkmeia churashimaensis* (AB548955)	0/2/570	99.6	*Dirkmeia churashimaensis*
DMKU-RP105	AB920309	*Rhodotorula taiwanensis* (GU646863)	1/0/519	99.8	*Rhodotorula taiwanensis*
DMKU-RP106	LC498289	*Meyerozyma caribbica* (AY187283)	0/0/483	100	*Meyerozyma caribbica*
DMKU-RP107	LC498290	*Moesziomyces antarcticus* (KY108571)	0/2/572	99.7	*Moesziomyces antarcticus*
DMKU-RP108	LC498291	*Rhodotorula mucilaginosa* (AF070432)	1/1/532	99.6	*Rhodotorula mucilaginosa*
DMKU-RP109	AB922845	*Hannaella siamensis* (AB922844)	0/0/557	99.6	*Hannaella siamensis*[a]
DMKU-RP111	LC498292	*Dirkmeia churashimaensis* (AB548955)	0/0549	100	*Dirkmeia churashimaensis*
DMKU-RP112	LC498293	*Moesziomyces antarcticus* (KY108571)	0/0/562	100	*Moesziomyces antarcticus*
DMKU-RP113	LC498294	*Sakaguchia oryzae* (AY335161)	1/0/490	99.8	*Sakaguchia oryzae*
DMKU-RP116	LC498295	*Rhodotorula paludigena* (AF070424)	1/0/543	99.8	*Rhodotorula paludigena*
DMKU-RP117	LC498296	*Dirkmeia churashimaensis* (AB548955)	0/0/544	100	*Dirkmeia churashimaensis*
DMKU-RP118	LC498297	*Moesziomyces antarcticus* (KY108571)	0/0/568	100	*Moesziomyces antarcticus*
DMKU-RP119	AB922846	*Hannaella siamensis* (AB922844)	0/0/597	100	*Hannaella siamensis*[a]
DMKU-RP120	LC498298	*Papiliotrema rajasthanensis* (AM262324)	0/1/571	99.8	*Papiliotrema rajasthanensis*
DMKU-RP121	LC498299	*Candida parapsilosis* (U45754)	1/0/524	99.8	*Candida parapsilosis*
DMKU-RP122	LC498300	*Moesziomyces antarcticus* (KY108571)	0/1/558	99.8	*Moesziomyces antarcticus*
DMKU-RP123	LC498301	*Dirkmeia churashimaensis* (AB548955)	0/0/578	100	*Dirkmeia churashimaensis*
DMKU-RP125	LC498302	*Papiliotrema aspenensis* (KC469778)	0/1/505	99.8	*Papiliotrema aspenensis*
DMKU-RP126	LC498303	*Jaminaea angkorensis* (EU587489)	1/1/556	99.6	*Jaminaea angkorensis*
DMKU-RP127	LC498304	*Moesziomyces antarcticus* (KY108571)	0/1/567	99.8	*Moesziomyces antarcticus*
DMKU-RP128	AB920310	*Saitozyma flava*(AF075497)	0/0/581	100	*Saitozyma flava*
DMKU-RP129	LC498305	*Sakaguchia oryzae* (AY335161)	1/0/505	99.6	*Sakaguchia oryzae*
DMKU-RP130	LC498306	*Moesziomyces antarcticus* (KY108571)	0/0/553	100	*Moesziomyces antarcticus*
DMKU-RP132	LC498307	*Papiliotrema flavescens* (AB035042)	0/0/553	100	*Papiliotrema flavescens*
DMKU-RP133	LC498308	*Moesziomyces antarcticus* (KY108571)	0/0/579	100	*Moesziomyces antarcticus*
DMKU-RP134	LC498309	*Sporidiobolus pararoseus* (AF070437)	0/1/547	99.8	*Sporidiobolus pararoseus*
DMKU-RP135	LC498310	*Moesziomyces antarcticus* (KY108571)	0/0/580	100	*Moesziomyces antarcticus*
DMKU-RP137	LC498311	*Papiliotrema laurentii* (AF075469)	1/1/566	99.6	*Papiliotrema laurentii*
DMKU-RP138	LC498312	*Rhodotorula paludigena* (AF070424)	0/1/519	100	*Rhodotorula paludigena*
DMKU-RP140	LC498313	*Papiliotrema flavescens* (AB035042)	0/1/569	100	*Papiliotrema flavescens*
DMKU-RP142	LC498314	*Papiliotrema flavescens* (AB035042)	0/1/540	99.8	*Papiliotrema flavescens*
DMKU-RP144	LC498315	*Moesziomyces antarcticus* (KY108571)	0/0/553	100	*Moesziomyces antarcticus*
DMKU-RP146	LC498316	*Sporobolomyces blumeae* (AY213010)	1/0/542	99.8	*Sporobolomyces blumeae*
DMKU-RP147	LC498317	*Candida parapsilosis* (U45754)	0/0/519	100	*Candida parapsilosis*
DMKU-RP148	LC498318	*Papiliotrema japonica* (AF444760)	0/2/578	99.7	*Papiliotrema japonica*
DMKU-RP149	LC498319	*Dirkmeia churashimaensis* (AB548955)	0/1/554	100	*Dirkmeia churashimaensis*

Table S1. *continued.*

Yeast strain	GenBank accession no.	Closest species	Nucleotide substitutions/gap/ total nt	% identity	Identification
DMKU-RP150	LC498320	*Dirkmeia churashimaensis* (AB548955)	0/0/578	100	*Dirkmeia churashimaensis*
DMKU-RP151	LC498321	*Moesziomyces antarcticus* (KY108571)	1/0/581	99.8	*Moesziomyces antarcticus*
DMKU-RP152	LC498322	*Papiliotrema siamense* (AB909023)	0/0/556	100	*Papiliotrema siamense*
DMKU-RP153	LC498323	*Rhodotorula taiwanensis* (GU646863)	0/1/493	99.8	*Rhodotorula taiwanensis*
DMKU-RP154	LC498324	*Rhodotorula taiwanensis* (GU646863)	1/0/535	99.8	*Rhodotorula taiwanensis*
DMKU-RP155	LC498325	*Candida diddensiae* (U45750)	0/1/509	99.8	*Candida diddensiae*
DMKU-RP156	LC498326	*Papiliotrema japonica* (AF444760)	0/0/557	100	*Papiliotrema japonica*
DMKU-RP158	LC486511	*Rhodotorula mucilaginosa* (AF070432)	2/0/534	99.6	*Rhodotorula mucilaginosa*
DMKU-RP160	LC498327	*Papiliotrema japonica* (AF444760)	0/0/558	100	*Papiliotrema japonica*
DMKU-RP161	LC498328	*Moesziomyces antarcticus* (KY108571)	1/1/555	99.6	*Moesziomyces antarcticus*
DMKU-RP162	LC498329	*Meyerozyma caribbica* (AY187283)	1/0/521	99.8	*Meyerozyma caribbica*
DMKU-RP164	LC498330	*Dirkmeia churashimaensis* (AB548955)	0/1/578	99.8	*Dirkmeia churashimaensis*
DMKU-RP165	LC498331	*Papiliotrema japonica* (AF444760)	0/0/564	100	*Papiliotrema japonica*
DMKU-RP166	LC498332	*Rhodotorula taiwanensis* (GU646863)	1/0/535	99.8	*Rhodotorula taiwanensis*
DMKU-RP167	LC486505	*Moesziomyces antarcticus* (KY108571)	3/0/541	99.4	*Moesziomyces antarcticus*
DMKU-RP168	AB920311	*Meyerozyma caribbica* (AY187283)	2/0/510	99.6	*Meyerozyma caribbica*
DMKU-RP169	LC498333	*Candida parapsilosis* (U45754)	0/0/516	100	*Candida parapsilosis*
DMKU-RP170	LC498334	*Hannaella phetchabunensis* (AB922849)	0/0/553	100	*Hannaella phetchabunensis*
DMKU-RP172	LC498335	*Sporobolomyces blumeae* (AY213010)	0/1/548	99.8	*Sporobolomyces blumeae*
DMKU-RP173	LC498336	*Sporobolomyces blumeae* (AY213010)	0/0/542	100	*Sporobolomyces blumeae*
DMKU-RP174	LC498337	*Moesziomyces antarcticus* (KY108571)	1/1/470	99.6	*Moesziomyces antarcticus*
DMKU-RP176	LC498338	*Papiliotrema japonica* (AF444760)	0/0/603	100	*Papiliotrema japonica*
DMKU-RP177	LC498339	*Rhodotorula taiwanensis* (GU646863)	0/0/474	100	*Rhodotorula taiwanensis*
DMKU-RP178	LC498340	*Papiliotrema laurentii* (AF075469)	1/1/566	99.6	*Papiliotrema laurentii*
DMKU-RP180	LC498341	*Moesziomyces antarcticus* (KY108571)	0/0/545	100	*Moesziomyces antarcticus*
DMKU-RP182	LC498342	*Moesziomyces antarcticus* (KY108571)	1/0/553	99.8	*Moesziomyces antarcticus*
DMKU-RP183	LC498343	*Sporobolomyces blumeae* (AY213010)	0/0/520	100	*Sporobolomyces blumeae*
DMKU-RP184	LC498344	*Papiliotrema aspenensis* (KC469778)	1/0/514	99.8	*Papiliotrema aspenensis*
DMKU-RP186	LC498345	*Candida parapsilosis* (U45754)	2/0/524	99.6	*Candida parapsilosis*
DMKU-RP187	LC498346	*Rhodotorula taiwanensis* (GU646863)	0/1/536	99.8	*Rhodotorula taiwanensis*
DMKU-RP188	LC498347	*Papiliotrema japonica* (AF444760)	0/0/484	100	*Papiliotrema japonica*
DMKU-RP190	LC498348	*Sakaguchia oryzae* (AY335161)	0/1/563	99.8	*Sakaguchia oryzae*
DMKU-RP192	LC498349	*Hannaella siamensis* (AB922844)	0/0/571	100	*Hannaella siamensis*
DMKU-RP194	LC498350	*Papiliotrema japonica* AF444760)	0/0/550	100	*Papiliotrema japonica*
DMKU-RP195	LC498351	*Dirkmeia churashimaensis* (AB548955)	0/0/553	100	*Dirkmeia churashimaensis*
DMKU-RP196	LC498352	*Dirkmeia churashimaensis* (AB548955)	0/0/468	100	*Dirkmeia churashimaensis*
DMKU-RP198	LC498353	*Rhodotorula paludigena* (AF070424)	0/0/581	100	*Rhodotorula paludigena*
DMKU-RP199	LC498354	*Dirkmeia churashimaensis* (AB548955)	1/0/508	99.8	*Dirkmeia churashimaensis*

Table S1. *continued.*

Yeast strain	GenBank accession no.	Closest species	Nucleotide substitutions/gap/ total nt	% identity	Identification
DMKU-RP202	LC498355	*Moesziomyces antarcticus* (KY108571)	0/0/546	100	*Moesziomyces antarcticus*
DMKU-RP204	LC498356	*Moesziomyces aphidis* (AB089363)	0/0/333	100	*Moesziomyces aphidis*
DMKU-RP205	LC498357	*Moesziomyces antarcticus* (KY108571)	0/0/546	100	*Moesziomyces antarcticus*
DMKU-RP209	LC498358	*Moesziomyces antarcticus* (KY108571)	0/0/545	100	*Moesziomyces antarcticus*
DMKU-RP212	LC498359	*Moesziomyces antarcticus* (KY108571)	0/2/555	99.6	*Moesziomyces antarcticus*
DMKU-RP214	LC498360	*Moesziomyces antarcticus* (KY108571)	0/0/569	100	*Moesziomyces antarcticus*
DMKU-RP215	LC498361	*Hannaella pagnoccae* (FJ828959)	1/1/543	99.6	*Hannaella pagnoccae*
DMKU-RP218	LC498362	*Papiliotrema rajasthanensis* (AM262324)	1/0/553	99.8	*Papiliotrema rajasthanensis*
DMKU-RP220	LC498363	*Sakaguchia oryzae* (AY335161)	1/0/462	99.8	*Sakaguchia oryzae*
DMKU-RP221	LC498364	*Dirkmeia churashimaensi*s (AB548955)	0/0/519	100	*Dirkmeia churashimaensis*
DMKU-RP223	LC498365	*Dirkmeia churashimaensi*s (AB548955)	0/0/527	100	*Dirkmeia churashimaensis*
DMKU-RP224	LC498366	*Moesziomyces antarcticus* (KY108571)	0/0/469	100	*Moesziomyces antarcticus*
DMKU-RP227	LC498367	*Sporobolomyces carnicolor* (AY070008)	0/0/551	100	*Sporobolomyces carnicolor*
DMKU-RP231	LC498368	*Moesziomyces antarcticus* (KY108571)	1/1/555	99.6	*Moesziomyces antarcticus*
DMKU-RP232	LC498369	*Papiliotrema siamense* (AB909023)	0/0/556	100	*Papiliotrema siamense*
DMKU-RP233	AB920313	*Kodamaea ohmeri* (U45702)	1/0/447	99.8	*Kodamaea ohmeri*
DMKU-RP235	LC498370	*Rhodotorula taiwanensis* (GU646863)	0/0/509	100	*Rhodotorula taiwanensis*
DMKU-RP237	LC498371	*Dirkmeia churashimaensis* (AB548955)	0/0/570	100	*Dirkmeia churashimaensis*
DMKU-RP239	LC498372	*Moesziomyces antarcticus* (KY108571)	0/0/280	100	*Moesziomyces antarcticus*
DMKU-RP240	LC498373	*Sporobolomyces blumeae* (AY213010)	1/0/475	99.8	*Sporobolomyces blumeae*
DMKU-RP242	LC498374	*Jaminaea angkorensis* (EU587489)	0/0/452	100	*Jaminaea angkorensis*
DMKU-RP245	LC498375	*Dirkmeia churashimaensi*s (AB548955)	0/0/527	100	*Dirkmeia churashimaensis*
DMKU-RP246	LC498376	*Rhodotorula mucilaginosa* (AF070432)	1/0/498	99.8	*Rhodotorula mucilaginosa*
DMKU-RP248	LC498377	*Moesziomyces antarcticus* (KY108571)	1/1/458	99.6	*Moesziomyces antarcticus*
DMKU-RP251	LC498378	*Dirkmeia churashimaensis* (AB548955)	0/0/499	100	*Dirkmeia churashimaensis*
DMKU-RP252	LC498379	*Rhodotorula taiwanensis* (GU646863)	1/0/509	99.8	*Rhodotorula taiwanensis*
DMKU-RP253	LC498380	*Moesziomyces antarcticus* (KY108571)	0/0/436	100	*Moesziomyces antarcticus*
DMKU-RP254	LC498381	*Moesziomyces antarcticus* (KY108571)	0/0/400	100	*Moesziomyces antarcticus*
DMKU-RP258	LC498382	*Dirkmeia churashimaensis* AB548955)	0/0/439	100	*Dirkmeia churashimaensis*
DMKU-RP259	LC498383	*Rhodotorula taiwanensis* (GU646863)	1/1/535	99.6	*Rhodotorula taiwanensis*
DMKU-RP261	LC498384	*Moesziomyces antarcticus* (KY108571)	0/0/289	100	*Moesziomyces antarcticus*
DMKU-RP264	LC498385	*Papiliotrema japonica* (AF444760)	0/0/603	100	*Papiliotrema japonica*
DMKU-RP266	LC498386	*Sporidiobolus pararoseus* (AF070437)	0/0/513	100	*Sporidiobolus pararoseus*
DMKU-RP267	LC498387	*Rhodotorula taiwanensis* (GU646863)	1/0/489	99.8	*Rhodotorula taiwanensis*
DMKU-RP269	LC498388	*Meyerozyma guilliermondii* (U45709)	1/0/459	99.8	*Meyerozyma guilliermondii*
DMKU-RP273	LC498389	*Dirkmeia churashimaensi*s (AB548955)	0/0/530	100	*Dirkmeia churashimaensis*
DMKU-RP274	LC498390	*Sporobolomyces carnicolor* (AY070008)	0/0/449	100	*Sporobolomyces carnicolor*
DMKU-RP276	LC498391	*Papiliotrema nemorosus* (AF472625)	1/0/360	99.7	*Papiliotrema nemorosus*

Table S1. *continued.*

Yeast strain	GenBank accession no.	Closest species	Nucleotide substitutions/gap/ total nt	% identity	Identification
DMKU-RP279	LC498392	*Papiliotrema japonica* (AF444760)	0/0/536	100	*Papiliotrema japonica*
DMKU-RP280	LC498393	*Sakaguchia oryzae* (AY335161)	1/0/483	99.8	*Sakaguchia oryzae*
DMKU-RP281	LC498394	*Sporobolomyces carnicolor* (AY070008)	1/0/478	99.8	*Sporobolomyces carnicolor*
DMKU-RP282	LC498395	*Rhodotorula taiwanensis* (GU646863)	1/0/574	99.8	*Rhodotorula taiwanensis*
DMKU-RP293	LC498396	*Papiliotrema rajasthanensis* (AM262324)	0/0/499	100	*Papiliotrema rajasthanensis*
DMKU-RP295	LC498397	*Sporobolomyces carnicolor* (AY070008)	0/0/390	99.7	*Sporobolomyces carnicolor*
DMKU-RP297	LC498398	*Dirkmeia churashimaensi*s (AB548955)	0/0/389	100	*Dirkmeia churashimaensis*
DMKU-RP298	LC498399	*Papiliotrema flavescens* (AB035042)	0/0/428	100	*Papiliotrema flavescens*
DMKU-RP299	LC498400	*Hannaella siamensi*s (AB922844)	0/0/422	100	*Hannaella siamensis*
DMKU-RP300	LC498401	*Candida parapsilosis* (U45754)	0/0/567	100	*Candida parapsilosis*
DMKU-RP301	AB920314	*Rhodotorula paludigena* (AF070424)	0/0/399	100	*Rhodotorula paludigena*
DMKU-RP302	LC498402	*Sporobolomyces nakasei* (KY109757)	1/0/410	99.8	*Sporobolomyces nakasei*
DMKU-RP303	LC498403	*Moesziomyces antarcticus* (KY108571)	0/0/581	100	*Moesziomyces antarcticus*
DMKU-RP305	LC498404	*Rhodotorula taiwanensis* (GU646863)	1/1/473	99.6	*Rhodotorula taiwanensis*
DMKU-RP306	LC498405	*Papiliotrema flavescens* (AB035042)	0/0/339	100	*Papiliotrema flavescens*
DMKU-RP307	LC498406	*Candida parapsilosis* (U45754)	2/0/511	99.6	*Candida parapsilosis*
DMKU-RP309	LC498407	*Sporidiobolus pararoseus* (AF070437)	0/0/349	100	*Sporidiobolus pararoseus*
YE-9	LC498408	*Rhodotorula taiwanensis* (GU646863)	1/0/574	99.9	*Rhodotorula taiwanensis*
YE-11	LC498409	*Pseudozyma alboarmeniaca* (AB117961)	1/0/573	99.8	*Pseudozyma alboarmeniaca*
YE-12	LC498410	*Dirkmeia churashimaensis* (AB548955)	0/0/532	100	*Dirkmeia churashimaensis*
YE-14	LC498411	*Papiliotrema japonica* (AF444760)	0/0/603	100	*Papiliotrema japonica*
YE-31	LC498412	*Pseudozyma alboarmeniaca* (AB117961)	0/1/573	99.8	*Pseudozyma alboarmeniaca*
YE-32	LC486515	*Sporidiobolus pararoseus* (AF189977)	2/0/579	99.7	*Sporidiobolus pararoseus*
YE-33	LC498413	*Sporobolomyces blumeae* (AB279628)	0/0/541	100	*Sporobolomyces blumeae*
YE-36	LC498414	*Dirkmeia churashimaensis* (AB548955)	0/0/567	100	*Dirkmeia churashimaensis*
YE-37	LC498415	*Candida tropicalis* (U45749)	0/0/576	100	*Candida tropicalis*
YE-38	LC498416	*Moesziomyces antarcticus* (AB089359)	0/0/581	100	*Moesziomyces antarcticus*
YE-39	LC498417	*Dirkmeia churashimaensis* (AB548955)	1/0/531	99.8	*Dirkmeia churashimaensis*
YE-40	LC498418	*Moesziomyces antarcticus* (AB089359)	0/0/581	100	*Moesziomyces antarcticus*
YE-42	LC498419	*Wickerhamomyces anomalus* (KY110078)	0/0/530	100	*Wickerhamomyces anomalus*
YE-43	LC498420	*Papiliotrema siamense* (AB909023)	1/0/549	99.8	*Papiliotrema siamense*
YE-57	LC498421	*Moesziomyces antarcticus* (AB089359)	0/0/581	100	*Moesziomyces antarcticus*
YE-58	LC498422	*Hannaella sinensis* (NG_042362)	0/0/601	100	*Hannaella sinensis*
YE-59	LC498423	*Sporobolomyces blumeae* (AB279628)	0/0/512	100	*Sporobolomyces blumeae*
YE-60	LC486516	*Sporidiobolus pararoseus* (AF189977)	2/0/578	99.8	*Sporidiobolus pararoseus*
YE-61	LC498424	*Moesziomyces antarcticus* (AB089359)	0/0/578	100	*Moesziomyces antarcticus*
YE-62	LC498425	*Papiliotrema japonica* (AF444760)	0/0/603	100	*Papiliotrema japonica*
YE-70	LC498426	*Sporobolomyces blumeae* (AB279628)	0/0/579	100	*Sporobolomyces blumeae*
YE71	LC486513	*Dirkmeia churashimaensis* (AB548955)	3/2/569	99.1	*Dirkmeia churashimaensis*

Table S1. *continued.*

Yeast strain	GenBank accession no.	Closest species	Nucleotide substitutions/gap/ total nt	% identity	Identification
YE73	LC498427	*Moesziomyces antarcticus (*AB089359)	0/0/581	100	*Moesziomyces antarcticus*
YE-86	LC498428	*Ustilago siamensis* (AB117963)	0/0/573	100	*Ustilago siamensis*
YE-87	LC498429	*Papiliotrema japonica* (AF444760)	0/0/604	100	*Papiliotrema japonica*
YE-88	LC498430	*Rhodotorula taiwanensis* (GU646863)	0/0/574	100	*Rhodotorula taiwanensis*
YE-89	LC498431	*Moesziomyces antarcticus* (AB089359)	1/0/581	99.8	*Moesziomyces antarcticus*
YE-91	LC498432	*Dirkmeia churashimaensis* (AB548955)	0/0/567	100	*Dirkmeia churashimaensis*
YE-102	LC498433	*Rhodotorula mucilaginosa* (AF070432)	0/0/577	100	*Rhodotorula mucilaginosa*
YE-104	LC498434	*Moesziomyces antarcticus* (AB089359)	0/0/581	100	*Moesziomyces antarcticus*
YE-105	LC498435	*Papiliotrema aspenensis* (KC469778)	1/0/567	99.8	*Papiliotrema aspenensis*
YE-106	LC498436	*Ustilago siamensis* (AB117963)	0/0/575	100	*Ustilago siamensis*
YE-107	LC486517	*Sporidiobolus pararoseus* (AF189977)	3/0/577	99.5	*Sporidiobolus pararoseus*
YE-111	LC498437	*Candida tropicalis* (U45749)	0/0/539	100	*Candida tropicalis*
YE-112	LC498438	*Rhodotorula mucilaginosa* (AF070432)	0/0/540	100	*Rhodotorula mucilaginosa*
YE-113	LC498439	*Trichosporon insectorum* (AY520383)	0/0/589	100	*Trichosporon insectorum*
YE-114	LC498440	*Moesziomyces antarcticus* (AB089359)	0/0/574	100	*Moesziomyces antarcticus*
YE-116	LC498441	*Trichosporon asahii* (AF105393)	0/0/566	100	*Trichosporon asahii*
YE-121	LC498442	*Dirkmeia churashimaensis* (AB548955)	0/0/578	100	*Dirkmeia churashimaensis*
YE-122	LC498443	*Moesziomyces antarcticus* (AB089359)	0/0/611	100	*Moesziomyces antarcticus*
YE-124	AB922847	*Hannaella siamensis* (AB922844)	0/0/597	100	*Hannaella siamensis*[a]
YE-126	LC498444	*Rhodotorula taiwanensis* (GU646863)	0/0/574	100	*Rhodotorula taiwanensis*
YE-127	LC498445	*Papiliotrema flavescens* (AB035042)	0/0/545	100	*Papiliotrema flavescens*
YE-128	LC498446	*Kalmanozyma vetiver* (AB809649)	0/0/606	100	*Kalmanozyma vetiver*
YE-129	LC498447	*Sporobolomyces blumeae* (AB279628)	0/0/537	100	*Sporobolomyces blumeae*
YE-130	LC498448	*Moesziomyces antarcticus* (AB089359)	0/0/611	100	*Moesziomyces antarcticus*
YE-135	LC498449	*Papiliotrema japonica* (AF444760)	0/0/604	100	*Papiliotrema japonica*
YE-136	LC498450	*Dirkmeia churashimaensis* (AB548955)	0/0/575	100	*Dirkmeia churashimaensis*
YE-137	LC498451	*Rhodotorula paludigena* (AF070424)	0/0/581	100	*Rhodotorula paludigena*
YE-138	LC498452	*Sporobolomyces blumeae* (AB279628)	0/0/543	100	*Sporobolomyces blumeae*
YE-140	LC498453	*Moesziomyces antarcticus* (AB089359)	0/0/612	100	*Moesziomyces antarcticus*
YE-141	LC498454	*Papiliotrema japonica* AF444760)	0/0/567	100	*Papiliotrema japonica*
YE-142	LC498455	*Moesziomyces antarcticus* (AB089359)	0/0/612	100	*Moesziomyces antarcticus*
YE-144	LC498456	*Sporobolomyces blumeae* (AB279628)	0/0/544	100	*Sporobolomyces blumeae*
YE-145	LC498457	*Papiliotrema japonica* (AF444760)	0/0/603	100	*Papiliotrema japonica*
YE-147	LC498458	*Rhodotorula paludigena* (AF070424)	0/0/581	100	*Rhodotorula paludigena*
YE-148	LC498459	*Rhodotorula taiwanensis* (GU646863)	1/0/574	99.8	*Rhodotorula taiwanensis*
YE-149	LC498460	*Dirkmeia churashimaensis* (AB548955)	0/0/578	100	*Dirkmeia churashimaensis*
YE-151	LC498461	*Moesziomyces antarcticus* (AB089359)	0/0/611	100	*Moesziomyces antarcticus*
YE-153	LC498462	*Sporobolomyces blumeae* (AB279628)	0/0/578	100	*Sporobolomyces blumeae*

Table S1. *continued.*

Yeast strain	GenBank accession no.	Closest species	Nucleotide substitutions/gap/ total nt	% identity	Identification
YE-154	LC498463	*Blastobotrys arbuscula* (U40108)	2/0/544	99.6	*Blastobotrys arbuscula*
YE-155	LC486518	*Vishniacozyma taibaiensis* (AY557601)	8/0/602	98.7	Potential new species closest to *Vishniacozyma taibaiensis*
YE-156	AB922848	*Hannaella siamensis* (AB922844)	3/1/598	99.3	*Hannaella siamensis*[a]
YE-157	LC498464	*Dirkmeia churashimaensis* (AB548955)	0/0/578	100	*Dirkmeia churashimaensis*
YE-160	LC498465	*Papiliotrema flavescens* (AB035042)	1/0/570	99.8	*Papiliotrema flavescens*
YE-161	LC498466	*Jaminaea angkorensis* (EU587489)	1/1/592	99.7	*Jaminaea angkorensis*
YE-162	LC498467	*Dirkmeia churashimaensis* (AB548955)	0/0/578	100	*Dirkmeia churashimaensis*
YE-164	LC498468	*Hyphopichia burtonii* (U45712)	0/2/526	99.6	*Hyphopichia burtonii*
YE-165	LC498469	*Wickerhamomyces edaphicus* (AB436763)	2/6/573	98.6	*Wickerhamomyces edaphicus*
YE-166	LC498470	*Candida wangnamkhiaoensis* (AB682910)	1/0/553	99.8	*Candida wangnamkhiaoensi*
YE-169	LC498471	*Trichosporon asteroides* (AF075513)	1/0/569	99.8	*Trichosporon asteroides*
YE-170	LC006026	*Yamadazyma epiphylla* (LC006026)	0/0/518	100	*Yamadazyma epiphylla*[b]
YE-171	LC486514	*Rhodotorula mucilaginosa* (AF070432)	2/0/541	99.6	*Rhodotorula mucilaginosa*
YE-172	LC498472	*Moesziomyces antarcticus* (AB089359)	0/0/611	100	*Moesziomyces antarcticus*
YE-175	LC498473	*Rhodotorula mucilaginosa* (AF070432)	1/0/538	99.8	*Rhodotorula mucilaginosa*

[a] Kaewwichian et al. (2015)
[b] Jindamorakot et al. (2015)

Table S2. Sequence similarity analysis of the ITS region of corn phylloplane isolates showing ≥2 nucleotide substitutions in the D1/D2 region.

Strain	GenBank accession no. of D1/D2, ITS region	Closest species	Nucleotide substitutions/ gap/total nt		Identification
			D1/D2 region	ITS region	
YE-71	LC486513, LC486527	*Dirkmeia churashimaensis* OK96^T (AB548955, AB548947)	3/2/569	4/4/602	*Dirkmeia churashimaensis*
DMKU-RP167	LC486505, LC486519	*Moesziomyces antarcticus* CBS5955^T (KY108571, AB089358)	3/0/541	3/0/684	*Moesziomyces antarcticus*
DMKU-RP11	LC486506, LC486520	*Rhodotorula taiwanensis* CBS 11729^T (GU646863, NR_157462)	2/0/538	1/1/583	*Rhodotorula taiwanensis*
DMKU-RP37	LC486507, LC486521	*Rhodotorula taiwanensis* CBS 11729^T (GU646863, NR_157462)	2/1/503	1/0/573	*Rhodotorula taiwanensis*
DMKU-RP38	LC486508, LC486522	*Rhodotorula toruloides* CBS 349 (AF070426, AF444489)	3/1/559	1/0/578	*Rhodotorula toruloides*
DMKU-RP29	LC486509, LC486523	*Rhodotorula toruloides* CBS 349 (AF070426, AF444489)	5/0/556	5/4/584	Potential new species closest to *Rhodotorula toruloides*
DMKU-RP53	LC486510, LC486524	*Rhodotorula toruloides* CBS 349 (AF070426, AF444489)	5/0/557	5/4/585	Potential new species closet to *Rhodotorula toruloides*
DMKU-RP158	LC486511, LC486525	*Rhodotorula mucilaginosa* JCM 8115^T (AF070432, AF444541)	2/0/534	2/1/596	*Rhodotorula mucilaginosa*
YE-171	LC486514, LC486532	*Rhodotorula mucilaginosa* JCM 8115^T (AF070432, AF444541)	2/0/541	2/0/599	*Rhodotorula mucilaginosa*
DMKU-RP02	LC486512, LC486526	*Saitozyma flava* CBS 331^T (AF075497, AF444338)	2/1/575	3/0/498	*Saitozyma flava*
YE-32	LC486515, LC486528	*Sporidiobolus pararoseus* CBS 491^T (AF189977, NR_155770)	2/0/579	2/1/572	*Sporidiobolus pararoseus*
YE-60	LC486516, LC486529	*Sporidiobolus pararoseus* CBS 491^T (AF189977, NR_155770)	2/0/578	2/1/576	*Sporidiobolus pararoseus*
YE-107	LC486517, LC486530	*Sporidiobolus pararoseus* CBS 491^T (AF189977, NR_155770)	3/0/577	2/1/572	*Sporidiobolus pararoseus*
YE-155	LC486518, LC486531	*Vishniacozyma taibaiensis* CBS 9919^T (AY557601, NR_144810)	8/0/602	21/7/499	Potential new species closest to *Vishniacozyma taibaiensis*

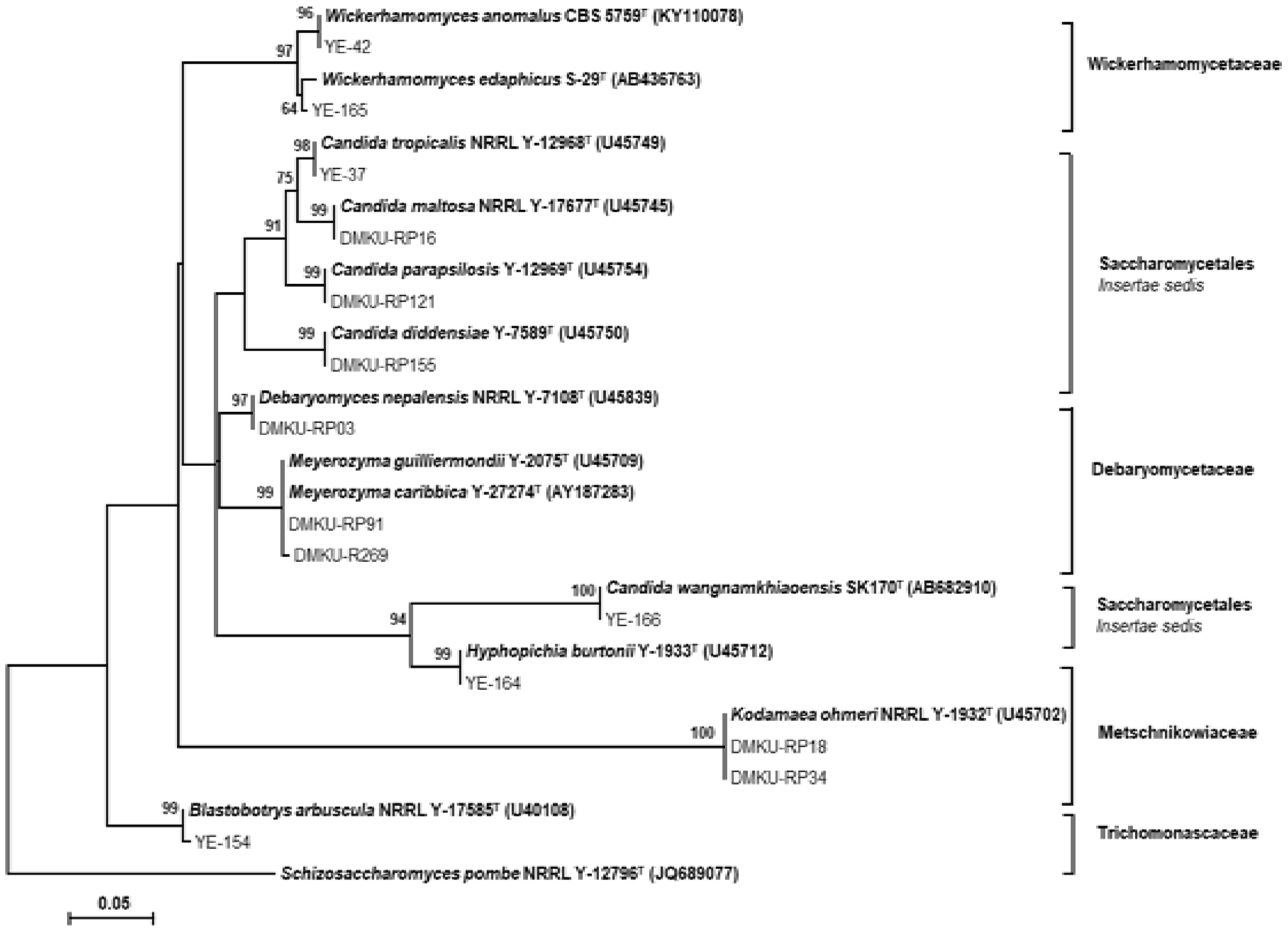

Figure S1. Phylogenetic relationship of the sequences of the D1/D2 region of the LSU rRNA gene of yeast strains isolated from the phylloplane of rice belonged to the Phylum Ascomycota, subphylum Saccharomycotina and their closely related yeast sequences retrieved from the GenBank database calculated from maximum-likelihood using GTR model. Number on branches represent the bootstrap percentage (>50%) from 1,000 random replications. The scale bar corresponds to a genetic distance of 0.05 substitutions per position.

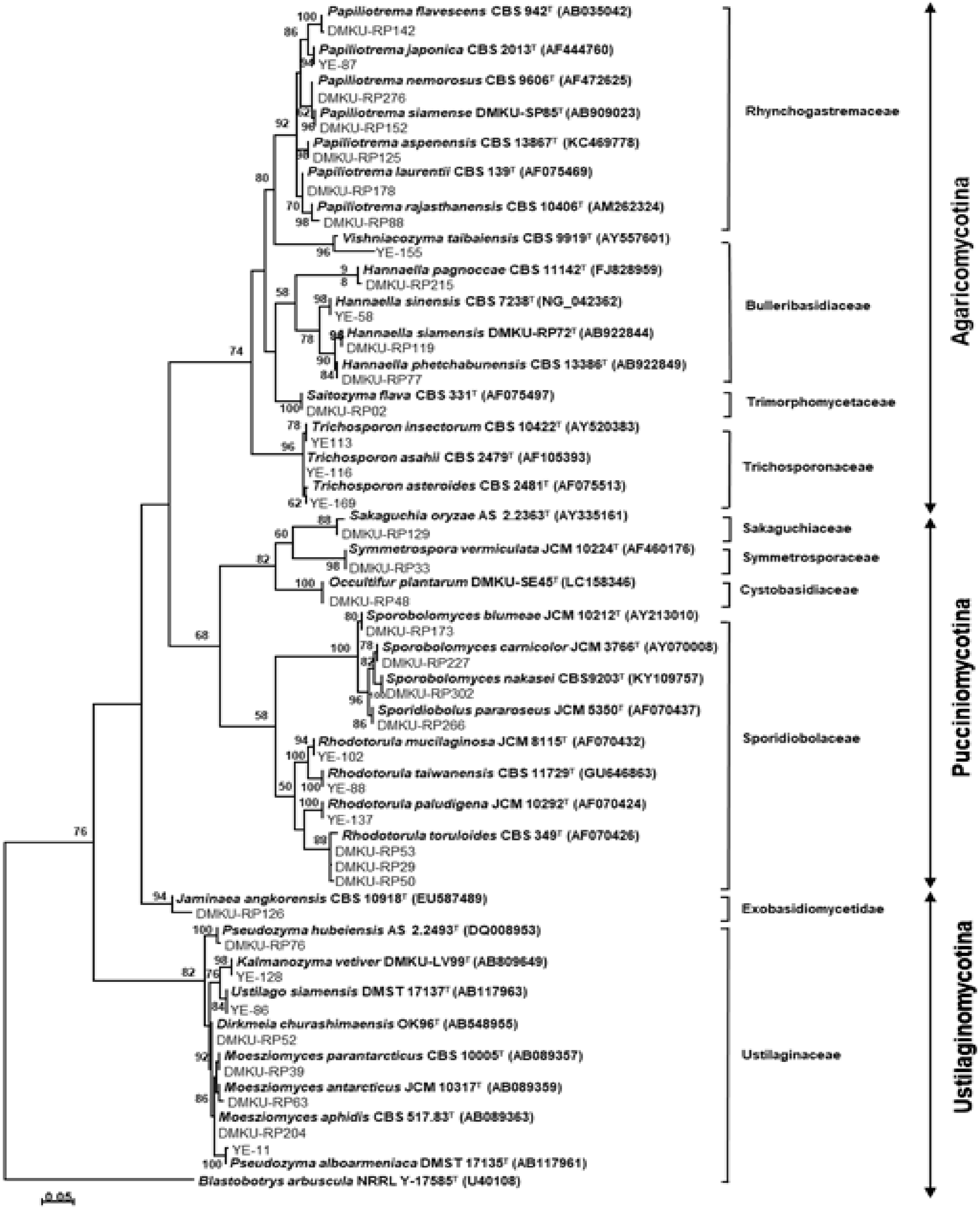

Papiliotrema flavescens CBS 942T (AB035042)
DMKU-RP142
Papiliotrema japonica CBS 2013T (AF444760)
YE-87
Papiliotrema nemorosus CBS 9606T (AF472625)
DMKU-RP276
Papiliotrema siamense DMKU-SP85T (AB909023)
DMKU-RP152
Papiliotrema aspenensis CBS 13867T (KC469778)
DMKU-RP125
Papiliotrema laurentii CBS 139T (AF075469)
DMKU-RP178
Papiliotrema rajasthanensis CBS 10406T (AM262324)
DMKU-RP88
Vishniacozyma taibaiensis CBS 9919T (AY557601)
YE-155
Hannaella pagnoccae CBS 11142T (FJ828959)
DMKU-RP215
Hannaella sinensis CBS 7238T (NG_042362)
YE-58
Hannaella siamensis DMKU-RP72T (AB922844)
DMKU-RP119
Hannaella phetchabunensis CBS 13386T (AB922849)
DMKU-RP77
Saitozyma flava CBS 331T (AF075497)
DMKU-RP02
Trichosporon insectorum CBS 10422T (AY520383)
YE113
Trichosporon asahii CBS 2479T (AF105393)
YE-116
Trichosporon asteroides CBS 2481T (AF075513)
YE-169
Sakaguchia oryzae AS 2.2363T (AY335161)
DMKU-RP129
Symmetrospora vermiculata JCM 10224T (AF460176)
DMKU-RP33
Occultifur plantarum DMKU-SE45T (LC158346)
DMKU-RP48
Sporobolomyces blumeae JCM 10212T (AY213010)
DMKU-RP173
Sporobolomyces carnicolor JCM 3766T (AY070008)
DMKU-RP227
Sporobolomyces nakasei CBS9203T (KY109757)
DMKU-RP302
Sporidiobolus pararoseus JCM 5350T (AF070437)
DMKU-RP266
Rhodotorula mucilaginosa JCM 8115T (AF070432)
YE-102
Rhodotorula taiwanensis CBS 11729T (GU646863)
YE-88
Rhodotorula paludigena JCM 10292T (AF070424)
YE-137
Rhodotorula toruloides CBS 349T (AF070426)
DMKU-RP53
DMKU-RP29
DMKU-RP50
Jaminaea angkorensis CBS 10918T (EU587489)
DMKU-RP126
Pseudozyma hubeiensis AS 2.2493T (DQ008953)
DMKU-RP76
Kalmanozyma vetiver DMKU-LV99T (AB809649)
YE-128
Ustilago siamensis DMST 17137T (AB117963)
YE-86
Dirkmeia churashimaensis OK96T (AB548955)
DMKU-RP52
Moesziomyces parantarcticus CBS 10005T (AB089357)
DMKU-RP39
Moesziomyces antarcticus JCM 10317T (AB089359)
DMKU-RP63
Moesziomyces aphidis CBS 517.83T (AB089363)
DMKU-RP204
YE-11
Pseudozyma alboarmeniaca DMST 17135T (AB117961)
Blastobotrys arbuscula NRRL Y-17585T (U40108)
0.05
Rhynchogastremaceae
Bulleribasidiaceae
Trimorphomycetaceae
Trichosporonaceae
Sakaguchiaceae
Symmetrosporaceae
Cystobasidiaceae
Sporidiobolaceae
Exobasidiomycetidae
Ustilaginaceae
Agaricomycotina
Pucciniomycotina
Ustilaginomycotina

Figure S2. Phylogenetic relationship of the sequences of the D1/D2 region of the LSU rRNA gene of yeast strains isolated from the phylloplane of rice belonged to phylum Basidiomycota and their closely related yeast sequences retrieved from the GenBank database calculated from maximum-likelihood using GTR model. Number on branches represent the bootstrap percentage (>50%) from 1,000 random replications. The scale bar corresponds to a genetic distance of 0.05 substitutions per position.
